# The coexistence of psychiatric and gastrointestinal problems in children with restrictive eating in a nationwide Swedish twin study

**DOI:** 10.1186/s40337-017-0154-2

**Published:** 2017-08-21

**Authors:** Jakob Täljemark, Maria Råstam, Paul Lichtenstein, Henrik Anckarsäter, Nóra Kerekes

**Affiliations:** 1 Lund University, Medical Faculty, Department of Clinical Sciences Lund, Child and Adolescent Psychiatry, Baravägen 1, S-221 85 Lund, Sweden; 2 Gillberg Neuropsychiatry Centre, University of Gothenburg, Gothenburg, Sweden; 3 Department of Medical Epidemiology and Biostatistics, Karolinska Institutet, Stockholm, Sweden; 4 CELAM (Centre for Ethics, Law and Mental Health), Institute of Neuroscience and Physiology, University of Gothenburg, Gothenburg, Sweden; 5 Department of Health Sciences, University West, Trollhättan, Sweden

**Keywords:** Restrictive eating problem, Neurodevelopmental problem, Psychiatric problem, Gastrointestinal problem, Twin study

## Abstract

**Background:**

Restrictive eating problems are rare in children but overrepresented in those with neurodevelopmental problems. Comorbidities decrease wellbeing in affected individuals but research in the area is relatively scarce. This study describes phenotypes, regarding psychiatric and gastrointestinal comorbidities, in children with restrictive eating problems.

**Methods:**

A parental telephone interview was conducted in 9- or 12-year old twins (*n* = 19,130) in the Child and Adolescent Twin Study in Sweden. Cases of restrictive eating problems and comorbid problems were established using the Autism, Tics-AD/HD and other Comorbidities inventory, parental reports of comorbidity as well as data from a national patient register. In restrictive eating problem cases, presence of psychiatric and gastrointestinal comorbidity was mapped individually in probands and their co-twin. Two-tailed Mann–Whitney U tests were used to test differences in the mean number of coexisting disorders between boys and girls. Odds ratios were used to compare prevalence figures between individuals with or without restrictive eating problems, and Fisher exact test was used to establish significance.

**Results:**

Prevalence of restrictive eating problems was 0.6% (concordant in 15% monozygotic and 3% of dizygotic twins). The presence of restrictive eating problems drastically increased odds of all psychiatric problems, especially autism spectrum disorder in both sexes (odds ratio = 11.9 in boys, odds ratio = 10.1 in girls), obsessive-compulsive disorder in boys (odds ratio = 11.6) and oppositional defiant disorder in girls (odds ratio = 9.22). Comorbid gastrointestinal problems, such as lactose intolerance (odds ratio = 4.43) and constipation (odds ratio = 2.91), were the most frequent in girls. Boy co-twins to a proband with restrictive eating problems generally had more psychiatric problems than girl co-twins and more girl co-twins had neither somatic nor any psychiatric problems at all.

**Conclusions:**

In children with restrictive eating problems odds of all coexisting psychiatric problems and gastrointestinal problems are significantly increased. The study shows the importance of considering comorbidities in clinical assessment of children with restrictive eating problems.

## Plain English Summary

Restrictive eating problems, where children do not get enough nutrition through their diet, are rare overall but more common in children with psychiatric problems. This study investigated which psychiatric and gastrointestinal problems (problems related to the stomach and the intestines) are also present, and how often, in children who have restrictive eating problems. We also studied what type of problems the twin-pair of a child with restrictive eating problems has.

We found that six in every 1000 children (9 or 12-year-olds) in a nation-wide general population had severe restrictive eating problems. The chance that both twins had this problem was much higher in identical (one egg) twins, suggesting that severe restrictive eating problems are partially inherited.

If a child had restrictive eating problems, risks were high that he/she also had other psychiatric problems. The risk for autism spectrum disorders was very high in girls and boys with restrictive eating problems. Even problems with digestion were frequent in these children, for example lactose intolerance and constipation were often occurring.

The results from this study show how important it is for health care professionals to assess for both psychiatric and gastrointestinal problems in children with severe restrictive eating problems.

## Background

In children and adolescents, a diagnosis of a formal eating disorder is at the extreme end of a broader spectrum of abnormal eating attitudes and behaviors [1]. Younger children often present with sub-threshold heterogeneous disordered eating symptoms [2] and relative to adolescents or adults, few children meet threshold criteria for specific eating disorders. New diagnostic categories introduced in the Diagnostic and Statistical Manual of Mental Disorders**,** 5th ed (DSM-5) [3] increase the number of individuals diagnosable with specific feeding and eating disorders by introduction of new diagnoses e.g. Avoidant/Restrictive Food Intake Disorder (ARFID) [2]. This study focuses on eating problems that differ from ARFID: included cases in this study are pre-pubertal children that have significant problems with restrictive eating as well as fear of gaining weight.

It is known that childhood restrictive eating is a risk factor for developing formal eating disorders including anorexia nervosa (AN) later on [4, 5]. In a previous study, using data from the Child and Adolescent Twin Study in Sweden (CATSS) study but from a different birth cohort, restrictive eating in pre-pubertal children was found to be more common in children with neurodevelopmental problems such as attention deficit hyperactivity disorder or autism spectrum disorder, particularly if the two conditions co-existed [6]. The present study investigates a wider range of psychiatric comorbidities than the original study, and explores physical comorbidity for children with restrictive eating problems. While links between restrictive eating problems (REP) and obsessive-compulsive disorder are well described for adolescents [7], there is less data on these patterns in children. Data on the association between REP and externalizing behavioral problems (oppositional defiant disorder and conduct disorder) are scarce for all ages.

Gastrointestinal symptoms are often reported in cases of formal eating disorders in children and adolescents, with constipation the most common complaint in cases of eating disorders with restrictive eating [8]. Gastrointestinal symptoms appear more common in ARFID than in AN or bulimia nervosa (BN) [9]. There are however few studies aiming to describe the wide range of comorbid gastrointestinal conditions to restrictive eating problems.

To better understand the function and symptom load in cases of clinically relevant eating problems it is essential to understand which other disorders coexist and what implications this overlap has for individuals. We hypothesised that middle school aged children with restrictive eating resulting in low weight would have an increased risk for adverse mental and somatic health problems compared to the rest of their peers. The aims of this study are twofold:

(1) To characterize the REP-phenotype by concurrently describing seven different possible coexisting psychiatric and five different gastrointestinal problems in probands.

(2) To compare the rates of the coexisting mental disorders and somatic disorders in REP-discordant monozygotic versus dizygotic co-twins, in order to illustrate the full range of phenotypical expressions of shared genetic and environmental effects.

## Methods

### Participants

The study participants were recruited from the ongoing longitudinal Child and Adolescent Twin Study in Sweden (CATSS) [10]. Data in this study was collected from the CATSS 9/12 sub-study launched in 2004. In CATSS 9/12, all parents of Swedish twins were invited to participate in a telephone interview regarding aspects of their children’s mental and physical health and social circumstances in connection with their children’s 9th (or 12th - for the first three years of the study only) birthdays. The overall response rate was 80% [10]. In 2013 the CATSS 9/12 was linked to the National Patient Register, which encompasses data on all Swedish psychiatric inpatient care since 1987 and since 2001 it also comprises information from outpatient consultations with specialist physicians.

Data from parental interviews performed with the birth cohorts between 1992 and 2000 of CATSS 9/12 was used in the present study. It included information on 19,130 children (51% boys), of whom 28.2% were monozygotic (MZ) twins, 35.3% dizygotic (DZ) same sex (ss) and 34.3% DZ different sex (ds) twins, while 2.2% had unknown zygosity. Zygosity was determined with the help of a panel of 49 single nucleotide polymorphisms using the children’s saliva samples [11]. When DNA was not available, a previously developed algorithm of questions was used. Only twins with >95% probability of being correctly classified were assigned zygosity by this method, and thus 2.2% (420 twins) were excluded on the basis that their zygosity was unknown, leaving 18,710 children. A further 43 children with chromosomal aberration (ICD-10 Q85, Q90–99) and 284 with brain damage (ICD-10 G00-G39, G45-G46, G80-G94, Q00-Q04) based on information from the National Patient Register were excluded resulting in a final study population of 18,383. For twin-pair analyses we further excluded twins without their co-twin, therefore those results are based on 9046 twin pairs (18,092 children). Among the 95 children with REP identified, five children were excluded as the data file did not contain information about their or their co-twins’ psychiatric and/or somatic health, leaving 90 children with REP for the co-twin analyses. Five of them were concordant pairs for REP, therefore co-twin analyses are presented for 85 probands with REP.

### Measures

REP was defined in two different ways, (a) through the eating module in the Autism, Tics-AD/HD and other Disorders (A-TAC) inventory, and (b) by direct questions to parent relating to earlier diagnosis of clinical eating disorder.

- *The Autism, Tics-AD/HD and other Disorders (A-TAC*) inventory: This inventory has been described in more detail elsewhere [12]. In short, it is a questionnaire developed for use in large-scale epidemiological research to investigate child psychiatric problems based on criteria stated in the Diagnostic and Statistical Manual of Mental Disorders**,** 4th ed, text rev. (DSM-IV-R) [13] and can be carried out as a parental phone interview. The eating module in A-TAC screens for restrictive eating problems and includes two questions: “has (s)he ever failed to gain enough weight for more than a year?” and “has (s)he seemed fearful of gaining weight or growing fat?”. Questions addressing specific symptoms or characteristics in A-TAC may be answered by the response categories “no” (score 0), “yes, to some extent” (score 0.5), and “yes” (score 1.0). REP by A-TAC was defined as scoring at least one and a half points (≥1.5) on the collapsed score for these two questions.
- *Restrictive eating problems diagnostic question in CATSS*: the CATSS telephone interview also contains two specific questions that are worded “has your child ever received a diagnosis of anorexia nervosa/ bulimia nervosa?” A positive answer to the first of these two questions qualified the child to be included in the REP group.

### Definition of coexisting disorders

Other coexisting disorders were defined via (i) the relevant ‘diagnostic module’ on the A-TAC using validated low cutoffs for each, or by (ii) recognition in the National Patient Register.

(i) *A-TAC diagnoses:* In the A-TAC, screening cutoffs with high sensitivity but lower specificity have been validated for Autism Spectrum Disorder (ASD), Attention Deficit and Hyperactivity Disorder (ADHD), Learning Disorder (LD), Tics Disorder (TD) [12] and conduct disorder (CD) [14]. For oppositional defiant disorder (ODD) a validated high cutoff [14] was used in the present study, because the low specificity of the low cutoff would result in an unreasonable high prevalence of ODD. Beside validity, the internal reliability of the scales has also been established previously with Cronbach’s α for ASD 0.86, ADHD 0.92, TD 0.57, ODD 0.75 and CD 0.61 [10]. Obsessive-Compulsive Disorder (OCD) has not been included in previous A-TAC validation studies [12, 14] [15]. However, the questions of the OCD module show good agreement with clinical practice [16].
(ii) *National patient register:* The National Patient Register encompasses data regarding clinical diagnoses from all psychiatric inpatient care since 1987 and since 2001 the register also comprises information from outpatient consultations with psychiatry specialist physicians. From the National Patient Register, information about ICD-10 diagnoses of ASD, ADHD, LD, TD, ODD, OCD and CD were also collected.

### Identification of somatic problems

The identified somatic problems were: celiac disease, lactose intolerance, other food or nutritional allergies (apart from celiac disease or lactose intolerance, from now on referred to as “food allergy”), diarrhea and constipation. The telephone interview questions regarding gastrointestinal conditions are straight forward yes or no questions, e.g. “does (s)he have or has (s)he ever had lactose intolerance (celiac disease; other food allergy)?” or “does (s)he have or has (s)he ever had problems with prolonged periods of diarrhea (constipation) growing up?”. Answers are coded for “yes” as “1” and for “no” as “0”, while the alternatives “do not know” or “do not wish to answer” are coded as missing values.

### Statistical methods

The statistical analysis was made using IBM SPSS Statistics version 22.

Among 85 children with REP available for twin pair analyses, there were five pairs where both twins had REP (concordant pairs for REP). In these pairs the proband was defined randomly. This resulted in 80 REP probands with known zygosity. For each of these probands and their co-twins coexisting problems were plotted separately for gender and for zygosity class. Concordance rate was defined as the percentage of co-twins meeting criteria for REP. Two-tailed Mann–Whitney U tests were used to test differences in the mean number of coexisting disorders between boys and girls.

The odds ratio (OR) for psychiatric problems and gastrointestinal problems was calculated for REP by comparison of the prevalence in this group to individuals without REP, and using Fisher exact test to establish significance.

## Results

### Prevalence of restrictive eating problems

In total, 95 children (40 boys, 55 girls) fulfilled criteria for REP corresponding to a population prevalence of 0.6% with a male–female ratio of 1 to 1.4. Of these 95 children 94 were identified from the A-TAC interview and 3 by parental reports about existing diagnosis of AN (2 of them by both). There were no parental reports about bulimic behavior.

### Coexisting problems in children with restrictive eating problems

Table 1 summarizes the prevalence of defined psychiatric and gastrointestinal problems in those boys and girls who were selected for REP. The most prevalent co-existing psychiatric problems were ADHD and LD (both present in almost 36% of children with REP), and the most prevalent co-existing gastrointestinal problems were constipation (in 22% of children with REP) followed by lactose intolerance and food allergies (each in about 12% of children with REP). Generally, there was a strong trend towards boys with REP having a higher prevalence of other psychiatric problems than girls with REP (mean composite number of psychiatric diagnoses 1.90 in boys, 1.09 in girls, *p* = 0.051) (Table 1). For specific conditions, significantly more boys than girls with REP had co-existing ASD, ADHD, TD or OCD. Coexisting gastrointestinal problems did not differ significantly between boys and girls.

**Table 1:** Prevalence and composite number of comorbid psychiatric and gastrointestinal problems in boys and girls with restrictive eating problems (REP) according to A-TAC (*n* = 95)

	REP (*n* = 95)	Boys with REP (*n* = 40)^a^	Girls with REP (*n* = 55)^b^	*p* of gender difference
ASD	22 (23.2%)	14 (35.0%)	8 (14.5%)	0.027
ADHD	34 (35.8%)	20 (50%)	14 (25.4%)	0.018
LD	34 (35.8%)	16 (40%)	18 (32.7%)	0.519
TD	10 (10.6%)	8 (20%)	2 (3.6%)	0.015
OCD	9 (9.5%)	7 (17.5%)	2 (3.6%)	0.033
ODD	14 (14.7%)	5 (12.5%)	9 (16.3%)	0.771
CD	15 (15.8%)	8 (20%)	7 (12.7%)	0.399
Composite^¤^ (SD)	1.45 (1.78)	1.90 (2.0)	1.09 (1.54)	0.051
Celiac disease	2 (2.1%)	0 (0.0%)	2 (3.6%)	0.506
Lactose intolerance	12 (12.6%)	5 (12.5%)	7 (12.7%)	1.00
Food allergy	11 (11.6%)	5 (12.5%)	6 (10.9%)	1.00
Constipation	21 (22.1%)	5 (12.5%)	16 (29.1%)	0.079
Diarrhea	5 (5.3%)	3 (7.5%)	2 (3.6%)	0.646
Composite* (SD)	0.49 (0.73)	0.43 (0.69)	0.53 (0.76)	0.505

*ASD* autism spectrum disorder, *ADHD* attention deficit hyperactivity disorder, *LD* learning disorder, *TD* tics disorder, *OCD* obsessive compulsive disorder, *ODD* oppositional defiant disorder, *CD* conduct disorder, *A-TAC* The Autism-Tics, AD/HD and other Comorbidities inventory

¤Composite: Mean number of assigned psychiatric diagnoses ASD, ADHD, LD, TD, OCD, ODD, CD

*Composite: Mean number of assigned gastrointestinal problems celiac disease, lactose intolerance, food allergy, constipation, diarrhea

^a^ *n* = 39 for composite measure of psychiatric problems because of missing information for a boy in TD; *n* = 37 for composite measure of somatic problems based on missing data for three boys in lactose intolerance, food allergy and diarrhea

^b^ *n* = 51 for composite measure of somatic problems based on missing information for four girls in celiac disease, lactose intolerance (2) and diarrhea

When defining the odds of having another psychiatric or gastrointestinal problem in a child with REP, the prevalence of defined psychiatric and gastrointestinal problems in the group of children with REP was compared to the prevalence found in children without REP (controls, *n* = 18,261) (Table 2). Generally, the odds significantly increased of all co-existing psychiatric problems in children (both boys and girls) with REP, except for girls to have with REP co-existing TD or OCD. The relative risk for a boy with REP to have another psychiatric problem was elevated three to twelvefold and most markedly elevated for the co-existence of ASD (OR = 11.9, *p* < 0.001) and OCD (OR = 11.6 *p* < 0.001). For a girl with REP the odds of having comorbid psychiatric problems was elevated two to tenfold, most markedly for the co-existence of ASD (OR = 10.1, *p* > 0.001). The odds for a girl with REP to have ODD was nine-fold increased (OR = 9.22, *p* > 0.001) compared to controls, and higher than for a boy with REP.

**Table 2:** Prevalence of psychiatric and gastrointestinal problems in the population of children with restrictive eating problems (REP) and in those without REP, separately presented by genders

	Boys without REP (*n* = 9305)	Boys with REP (*n* = 40)	Odds ratio	Girls without REP (*n* = 8956)	Girls with REP (*n* = 55)	Odds ratio
ASD	402 (4.3%)	14 (16.9%)	11.91 (6.17–22.9) *p* < 0.001	149 (1.7%)	8 (14.5%)	10.06 (4.67–21.65) *p* < 0.001
ADHD	1213 (13.0%)	20 (31.5%)	6.66 (3.57–12.41) *p* < 0.001	576 (6.4%)	14 (25.5%)	4.96 (2.69–9.15) *p* < 0.001
LD	1557 (16.7%)	16 (28.1%)	3.32 (1.76–6.26) *p* < 0.001	1130 (12.6%)	18 (32.7%)	3.37 (1.91–5.94) *p* < 0.001
TD	412 (4.4%)	8 (13.6%)	5.57 (2.54–12.19) *p* < 0.001	154 (1.7%)	2 (3.6%)	2.16 (0.52–8.93) *p* = 0.247
OCD	167 (1.8%)	7 (11.2%)	11.61 (5.96–26.61) *p* < 0.001	126 (1.4%)	2 (3.6%)	2.65 (0.64–10.97) *p* = 0.184
ODD	322 (3.5%)	5 (12.5%)	3.98 (1.55–10.23) *p* = 0.001	186 (2.1%)	9 (16.4%)	9.22 (4.45–19.10) *p* < 0.001
CD	465 (5.0%)	8 (20.0%)	4.75 (2.18–10.36) *p* = 0.001	262 (2.9%)	7 (12.7%)	4.84 (2.17–10.79) *p* = 0.001
Celiac disease	78 (0.8%)	0 (0%)	0	125 (1.4%)	2 (3.6%)	2.71 (0.65–11.25) *p* = 0.177
Lactose intolerance	535 (5.7%)	5 (12.5%)	2.39 (0.93–6.13) *p* = 0.074	449 (5.0%)	7 (12.7%)	2.91 (1.30–6.49) *p* = 0.016
Food allergy	811 (8.7%)	5 (12.5%)	1.53 (0.60–3.92) *p* = 0.387	721 (8.1%)	6 (10.9%)	1.39 (0.59–3.26) *p* = 0.452
Constipation	543 (5.8%)	5 (12.5%)	2.30 (0.90–5.89) *p* = 0.083	756 (8.4%)	16 (29.1%)	4.43 (2.46–8.00) *p* < 0.001
Diarrhea	357 (3.8%)	3 (7.5%)	2.08 (0.64–6.79) *p* = 0.190	233 (2.6%)	2 (3.6%)	1.44 (0.35–5.93) *p* = 0.653

*ASD* autism spectrum disorder, *ADHD* attention deficit hyperactivity disorder, *LD* learning disorder, *TD* tics disorder, *OCD* obsessive compulsive disorder, *ODD* oppositional defiant disorder, *CD* conduct disorder

Boys were generally less affected by gastrointestinal problems than girls. In a girl with REP the odds were almost tripled for co-existing lactose intolerance (OR = 2.91 *p* < 0.001) and more than fourfold increased for constipation (OR = 4.43, *p* < 0.001).

### Psychiatric and somatic problems in co-twins of probands with restrictive eating problems

Three of 20 MZ pairs (15%) were concordant for REP (10% MZ boy, 20% MZ girl), while in DZ there were 2 of 65 pairs (3.1%) where both twins had REP (0% DZss boy, 0%DZss girl, 9% DZds co-twin girl, 0% DZds co-twin boy).

**Fig. 1:**
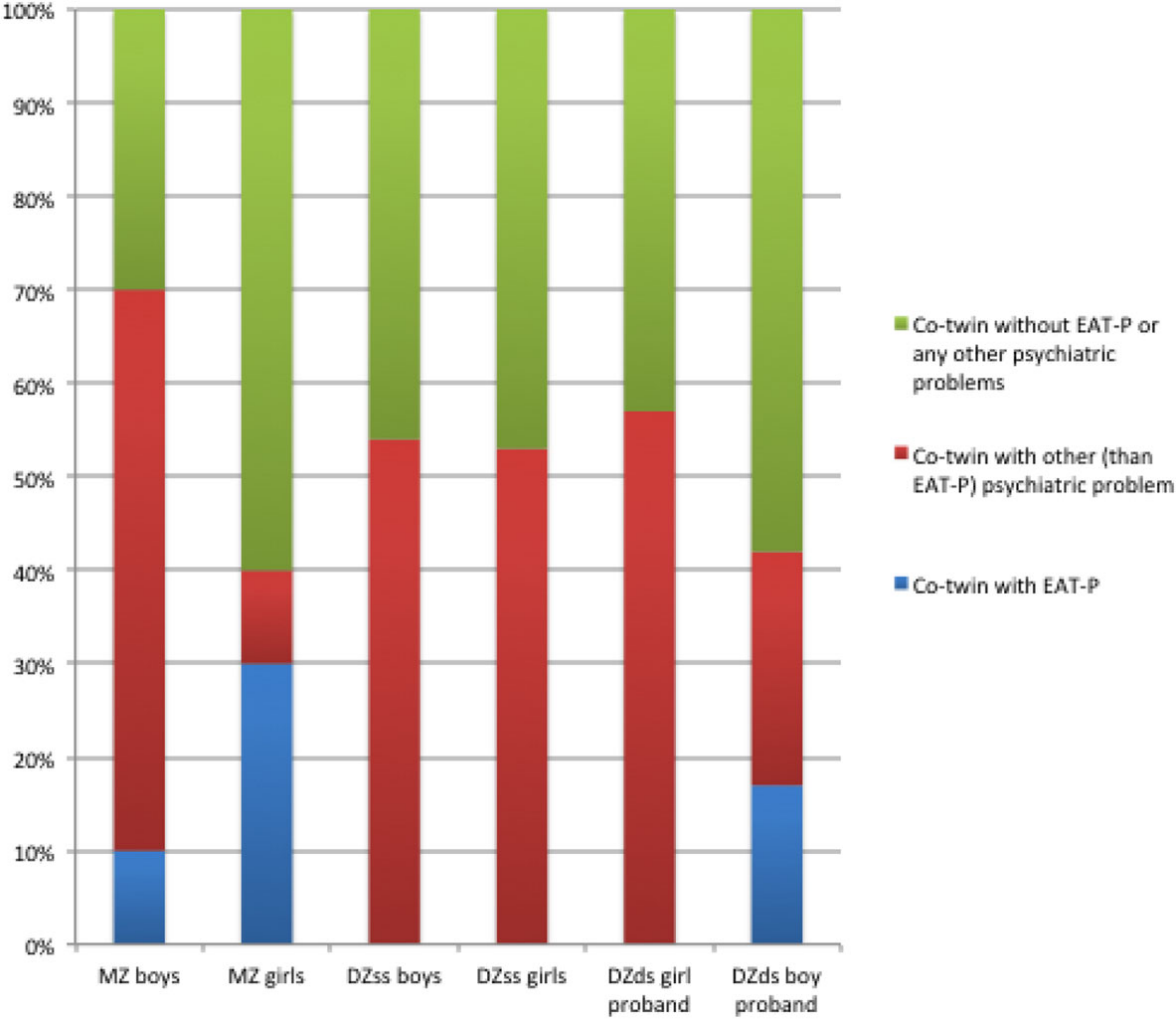
Sibling recurrence rates of REP and other psychiatric problems in co-twins of probands with REP according to proband zygosity (*MZ* monozygotic; *DZ* dizygotic) and gender. Note: other psychiatric problems included: ASD, ADHD, LD, TD, OCD, ODD and CD

Girl co-twins to a proband with REP more often had REP than a boy co-twin. Boy co-twins however more often had other psychiatric problems (ADHD, ASD, LD, TD, OCD, ODD or CD). 70% of MZ boy co-twins and 40% of MZ girl co-twins to a proband with REP had some psychiatric problem (REP and/or other psychiatric problem), leaving 30% of MZ boy and 60% of MZ girl co-twins without any psychiatric problem (Fig. 1). Around half of the DZ co-twins (43–58%) had no psychiatric problems (no REP and no other psychiatric problems) (Fig. 1).

One of four co-twins to a proband with REP had somatic problems. This prevalence was five times higher than the occurrence of somatic problems in control children (5%) and two and a half time higher than it was in probands with REP (9.8%). Similar proportions of boy and girl co-twins had (i) somatic problems, (ii) somatic problems only (i.e. no psychiatric problems) and (iii) neither psychiatric nor somatic complaints (around 40%) (Table 3).

**Table 3:** Prevalence of somatic problems in co-twins of probands with restrictive eating problems (REP)

	% of co-twins with somatic problems	% of co-twins with only somatic problems^a^	% of co-twin without somatic or psychiatric problems^b^
Boys *n* = 35	24.3	5.3	39.3
Girls *n* = 50	26.3	6.7	43.3

^a^Only somatic problem but no REP or any other psychiatric problems

^b^Those without any somatic problems or REP or psychiatric problem

## Discussion

### Summary of main findings

The prevalence of REP was 0.6% with a male to female ratio of 1 to 1.4. Individuals with REP had a higher prevalence of psychiatric problems than controls. The odds of comorbidity were increased for all studied psychiatric conditions in boys, most markedly for ASD and OCD, while in girls for all psychiatric conditions except TD and OCD, with most marked elevations in ASD and ODD. The odds ratio of constipation and lactose intolerance was elevated in girls with REP compared to controls. Concordance for REP was higher in MZ than in DZ co-twins. Co-twin girls were more often concordant for REP itself, while co-twin boys more often had other comorbid psychiatric problems.

### Restrictive eating problems in a nationwide general population of pre-pubertal children

The prevalence of restrictive eating problems in this sample was 0.6%. This prevalence is higher than previously reported, with one large British study quoting a prevalence of formal eating disorders of 0.15% among 11–12 year olds [17]. The higher prevalence in our study is a result of A-TAC not being a diagnostic instrument for formal eating disorders. Instead it recognizes children at risk: those whose parents report restrictive eating behaviors that potentially could lead to serious disorders. The prevalence figures in the current study of a non-clinical population may also reflect the fact that many youths with eating problems do not access treatment or receive a diagnosis, and that a high burden of disorders is likely to exist at a non-clinical sample level [10].

In this young study population, there was 1.4 times more girls than boys with REP. The higher proportion of REP cases in girls compared to boys is in agreement with findings of previous studies (for a review see [18]). Formal eating disorders have previously been described as far more common in female adolescents and adults than in males, with a rate ratio of lifetime prevalence of anorexia nervosa and bulimia nervosa in males versus females reported to be equal to or less than 1 to 10 [19]. However more recent studies utilizing DSM-5 criteria have shown a more equal sex distribution. A study of a Canadian adolescent community sample [20] showed that 2.2% of males and 4.5% of females met formal DSM-5 criteria for an eating disorder, and a further 1.1% of males and 5.1% of females met subthreshold criteria for eating disorders. Adolescent boys are also more likely to present with eating disorders other than formal AN or BN, such as atypical AN [21]. It has been suggested that gender differences in presentations, as well as gender specific differences in social constructions around weight ideals and eating habits, contribute to fewer clinically diagnosed cases of eating disorders in young males [21].

The present study assesses for eating disorders that include fear of gaining weight and weight loss/lack of weight gain for at least a year, conditions that put the individual at risk of developing AN or BN. It does not investigate eating problems without weight concerns, e.g. ARFID, or for binge eating disorder. Should these conditions have been investigated in this study it is likely that a more equal gender distribution would have been seen in this young age group. Fisher et al. [9] have established that ARFID occurs at an earlier age than AN or BN (12.9 years vs. 15.6 years vs 16.5 years) and that ARFID is more common in males than AN or BN, though female cases still dominate the ARFID category.

### Co-existing psychiatric problems in probands

The findings of the present study emphasize that psychiatric problems should be considered in children with REP and vice versa. One in three boys and one in four girls with REP screened positive for ADHD in our general population sample. Evidence is gathering that ADHD is common among children with eating problems [22], more markedly in boys than in girls, and especially if ADHD and ASD symptoms coexist [6] which they commonly do. One in six boys and girls with REP screened positive for ASD. Feeding problems are overrepresented in children with ASD [23] and consequential extreme low and high BMIs have been observed in ASD, potentially as an effect of stereotyped or repetitive behaviors [24].

Our study indicates a strong trend towards boys with REP having a higher prevalence of psychiatric comorbidities than girls with REP (mean composite number of psychiatric comorbid psychiatric diagnoses 1.90 in boys, 1.09 in girls, *p* = 0.051). This overrepresentation is consistent with previous findings regarding gender differences in eating disorders [19]. The increased risk of REP and comorbid learning difficulties also mirrors previous findings. Some degree of problematic eating is evident in nearly all children with intellectual disability whether mild, moderate or severe [25] and eating disorders seem to be overrepresented in adult institutionalized patients with intellectual disability and to a lesser degree in adult individuals with intellectual disability living at home [26, 27].

There was an eleven-fold increased odds ratio of OCD in boys with REP in our study, while the twofold increased odds ratio for girls did not reach statistical significance. Eating disorders are known to be associated with OCD [28, 29] and in adult populations one third of patients with AN or BN have a lifetime diagnosis of OCD which more often than not precedes the debut of AN [30]. There are significant gender differences in the lifetime prevalence of AN and BN among patients with OCD [31] with marked female overrepresentation, while in this study boys with REP had more OCD than girls. The earlier debut of OCD in boys than in girls overall [31] could be a possible explanation for the gender difference on display in this sample.

Our sample displayed markedly increased odds of ODD and CD in both boys and girls with REP. There is a relative scarcity of research of links between REP and ODD or CD. One study showed that in a lifetime perspective, adults with BN and binge eating disorder, but not AN, had an increased risk of a lifetime diagnosis of ODD and CD [22] and a more recent study showed that children with restrictive or selective eating had higher rates of externalizing and oppositional behaviors [32]. ODD and CD often co-exists with other neurodevelopmental problems, most notably ADHD. Whether ODD or CD in isolation would increase risk of REP was not assessed in the present study.

### Co-existing gastrointestinal problems in probands

Gastrointestinal complaints are common in pre-pubertal children in general and more so in children who have formal eating disorders [9]. It is well established that there is a strong overlap between gastrointestinal symptoms and psychiatric disorders (for a review see e.g. Korterink et al. [33]). Furthermore, before pubertal age, psychiatric problems often present with somatic symptoms. In general, girls are known to have more functional complaints including functional abdominal pain [33] than boys. Pain is a common symptom in gastrointestinal disorders including lactose intolerance and constipation, and both sex-determined biological differences in pain thresholds and a gender-influenced greater willingness to report somatic experiences e.g. pain can be at play [34].

Constipation is a common problem in childhood (0.7–29%, median 8.9 (5.3–17.4)) [35]. It is typically characterized by infrequent bowel evacuations, large stools, and difficult or painful defecation [36]. In this study, we found increased odds of constipation for girls but not boys with REP. Evidence regarding gender differences in prevalence of childhood constipation in the general population is conflicting, with five of seven studies in a systematic review showing no difference and two studies showing overrepresentation in girls [35]. Constipation has previously been identified as a risk factor for disordered eating in an Israeli study of female adolescents [37]. Constipation in patients with AN is associated with slowed colonic transit time and this delay in transit may contribute to bloating, thus further exacerbating patients’ fear of fatness [38]. Consequent rectal distension could also reflexively inhibit gastric emptying, thus slowing down transit times further [38]. Constipation in children could be a consequence of reduced food intake generally. It is worsened by dehydration [39], and diet poor in fiber and nutrients [40], conditions for which children with REP may be at risk. Further, in AN poor muscle strength as a consequence of atrophy may contribute to constipation [41].

Lactose intolerance is a clinical syndrome where ingestion of lactose or lactose-containing food substances gives one or more of the following symptoms: abdominal pain, diarrhea, nausea, flatulence, and bloating [42]. Girls with REP had a twice increased risk of lactose intolerance and to our knowledge such a link has not previously been reported.

Prolonged problems with diarrhea are common complaints in pediatric practice. It is a symptom with several potential causes including infections and antibiotics treatment, irritable bowel syndrome, lactose intolerance and celiac disease for older children [43]. Chronic diarrhea refers to the persistence of loose stools (generally with increase in stool frequency) for at least 14 days [43]. The risk of diarrhea is known to be increased in children with developmental problems e.g. ASD [44]. We did not see any increased odds for diarrhea in children with REP which may seem surprising, given that problems with diarrhea are frequent in certain types of adolescent eating disorders e.g. AN purging subtype [45] and have been reported as a presenting complaint in children with ARFID presenting to pediatric services [46]. However, it is likely that restrictive eating behaviors in this population predispose more to constipation than to diarrhea. Overall, gastrointestinal symptoms are more common in patients with ARFID compared to cases of AN and BN [9]. If cases of ARFID were also screened for in this study, it is likely that the frequencies of gastrointestinal comorbidity would have been higher.

The high frequencies of gastrointestinal comorbidities in cases of REP should be viewed in the light of comorbidity figures for other psychiatric conditions, amongst which anxiety disorders are the most prevalent in younger age groups. In one study of 42 pediatric patients with functional chronic abdominal pain, 43% met criteria for depression and 79% for at least one anxiety disorder at the time of assessment [47], though it should be considered when interpreting these results that several anxiety disorder criteria according to DSM-IV-R [13] include somatic symptoms [48]. Another prospective study [49] of 332 pre-adolescent cases with functional abdominal pain compared to controls found elevated odds ratios of lifetime anxiety disorder (OR = 4.9) and anxiety disorder at time of follow up (OR = 3.6) in early adulthood. This study also showed that anxiety problems often persisted at follow up, even when gastrointestinal complaints had resolved. Egger, Costello, Erkanli and Angold [50] found in their population study of 9–16-year-old children that somatic complaints were strongly associated with emotional disorders in girls and with disruptive behaviors in boys. Specifically, boys with stomach aches had an increased OR of comorbid ODD (3.6) and ADHD (3.5). Interestingly, for girls an elevated OR for stomach aches comorbid to anxiety disorder was only seen in combination with another somatic complaint (headaches or musculoskeletal pain) in that study.

### Description of co-twins of probands with restrictive eating problems

Eating disorders are known to be at least moderately heritable. The heritability of AN was reported at 56% in a large genetic review [51] while Thornton, Mazzeo, & Bulik [52] quote heritability estimates for BN at 54–83% and binge eating disorder at 41–57%. Our findings that concordant REP is more often found in MZ than in DZ twins seems to strengthen previous findings about the importance of genetic background factors. There was a higher concordance rate found for REP in girls than in boys. These findings suggest a gender difference in the heritability pattern of REP and support the hypothesis formulated by Strober et al. [53] that eating disorders in men are more serious and require a greater loading of genes or adverse familial environmental factors for its expression compared to that in females.

Eating disorders have been suggested to be neurodevelopmental conditions [54]. In our study, there was a considerable overlap between REP probands and their co-twins with regards to either concordant REP, or co-occurring REP and neurodevelopmental problems. These findings suggest that REP potentially should be covered under the broad, thorough and holistic multidisciplinary assessments of Early Symptomatic Syndromes Eliciting Neurodevelopmental Clinical Examinations (ESSENCE) as suggested by Gillberg [55].

### Comorbidity - clinical and public health implications

There is not one single given definition of comorbidity generally [56] or in the area of child neurodevelopmental problems specifically [55], but comorbidity is here, as often, defined as co-existence of at least two separate diagnosed disorders whether or not they share a common pathophysiology. In the clinic, there is significant overlap in symptoms between different disorders, in the somatic as well as the psychiatric field, and at least in the neurodevelopmental field, children often move across diagnostic categories over time [55].

In general terms and for a range of conditions, the presence of comorbidities has been associated with multiple negative outcomes, including worse psychological and somatic health outcomes per se, and reduced quality of life and impaired overall function [48, 56].

For health care practitioners challenges include more complex clinical management [56], with increasing demands on interface with other agencies, and an increased risk of complications e.g. polypharmacy. The presence of comorbidities is also associated with costs that increase exponentially as the number of chronic conditions increases [56].

The challenges of comorbidity and their persistence thus call for well worked out and integrated pathways between family doctors, pediatricians and child psychiatrists and consideration of establishing multidisciplinary teams branching these specialties for children with significant eating problems.

### Limitations

This study has some important limitations. As previously described [57], the screening diagnosis of REP in the current version of A-TAC has not been formally validated. However, the criteria in the interview to get a screening diagnosis of REP are strict: a combination of weight loss/lack of weight gain for a whole year and having expressed fear of gaining weight or growing fat is required, making screening cases likely not only to be clinically significant but also indicating individuals at risk for developing formal eating disorders if they do not already have them [11].

An obvious limitation is that this is not a clinical sample, and individuals were identified as having gastrointestinal problems with parental report rather than by physician diagnosis. Parents generally have a fairly good knowledge about existence of children’s gastrointestinal symptoms but not necessarily the exact nature of the problem [58, 59].

As a cross-sectional study, this research establishes association between REP and somatic / psychiatric symptoms. It does not however examine causality of symptoms. Further research is warranted to examine symptom development and the pathological processes at play.

Further, the study population utilized was twins and this means that absolute prevalence numbers should be interpreted with some caution.

Finally, despite drawing on a large number of study participants, REPs are relatively uncommon in pre-pubertal children. This has a limiting effect of the power to detect significant differences between REP cases and controls.

## Conclusions

Restrictive eating problems increased the odds of comorbidities for all psychiatric problems studied, in particular for autism spectrum disorders. Girls with restrictive eating problems had increased odds of having lactose intolerance and constipation. The heterogeneous eating problems are often resistant to treatment, especially if underlying neurodevelopmental problems are neglected. A transdiagnostic approach will be useful to the development of characterisation, diagnostic formulation, and targeted intervention in eating disorders.

The study shows the importance of considering comorbidities in clinical assessment of children with restrictive eating problems.

## Abbreviations

ADHD: Attention deficit / hyperactivity disorder
AN: Anorexia nervosa
ARFID: Avoidant/restrictive food intake disorder
ASD: Autism spectrum disorder
A-TAC: The Autism-Tics, AD/HD and other Comorbidities inventory
BN: Bulimia nervosa
CATSS: Child and adolescent twin study in Sweden
CD: Conduct disorder
ds: Different sex
DSM-5: Diagnostic and Statistical Manual of Mental Disorders, 5th ed
DSM-IV-R: Diagnostic and Statistical Manual of Mental Disorders, 4th ed. revised
DZ: Dizygotic
LD: Learning disorder
MZ: Monozygotic
OCD: Obsessive-compulsive disorder
ODD: Oppositional defiant disorder
OR: Odds ratio
REP: Restrictive eating problems
ss: Same sex
TD: Tics disorder
